# Diet culture on TikTok: a descriptive content analysis

**DOI:** 10.1017/S1368980024001381

**Published:** 2024-09-23

**Authors:** Emily Munro, Gabriella Wells, Rigel Paciente, Nicole Wickens, Daniel Ta, Joelie Mandzufas, Karen Lombardi, Alix Woolard

**Affiliations:** 1 The Kids Research Institute Australia, Nedlands, WA 6009, Australia; 2 The University of Western Australia, Crawley, WA 6009, Australia; 3 Edith Cowan University, Joondalup, WA 6027, Australia

**Keywords:** Diet, TikTok, Eating disorders, Eating behaviours, Social media, Content analysis, Dieting, Body image, Adolescents

## Abstract

**Objective:**

To investigate how dieting is portrayed on TikTok and the potential implications for public health considering the effect of diet culture on eating disorders amongst young people.

**Design:**

A cross-sectional descriptive content analysis of 250 videos from the five most popular diet-related hashtags. A codebook was developed to analyse the content of the videos and collect the engagement for each video (likes, comments and shares).

**Setting:**

TikTok website.

**Participants:**

There were no participants in this study.

**Results:**

More than half of the videos portrayed ‘body checking’, a potentially harmful practice for body image. Of the videos that represented body image, almost half represented body image negatively. However, most videos promoted the idea of ‘healthy eating’, and only 6·4 % displayed disordered eating behaviours. Over half of the videos provided dietary advice, and of those videos, most content creators claimed to be experts (64 %). Claiming expertise was not significantly associated with engagement; however, the use of humour was significantly associated with engagement. Additionally, males were more likely than females to disclose their goals of the diet.

**Conclusions:**

Overall, it appears that TikTok is commonly used to share nutrition tips and personal experiences around dieting and eating in general, often employing humour as an effective technique. The popularity of the platform and rapid dissemination of information would be a useful tool for health professionals, especially those working with eating disorders, to utilise.

Eating behaviours are established in childhood through a variety of factors such as genetics, personal preferences, observational learning, socioeconomic status, food availability and cultural and social norms^(1)^. Social media also has a strong influence on the eating behaviours of young people, especially with the popularity of ‘influencers’ who are perceived as role models^(2,3)^. Children and adolescents closely watch the behaviours of important peers and adults in their lives, including admired social media influencers, and mimic their behaviours^(2,4)^. Companies employ influencer marketing on Instagram to promote their products through these trusted influencers, who reinforce the idea that buying certain products will enable you to change your body and make you healthy, happy and beautiful^(5)^. Children are also more likely to consume food and drinks produced by brands they have engaged with online, especially through online videos^(6)^. Social media may influence eating behaviours by creating or reinforcing existing social and cultural norms, with influence from peers and commercial entities^(7)^. Adolescents are more likely to participate in dieting if they have body dissatisfaction, low self-esteem, low sense of control over their life and mental health issues such as depression or anxiety^(8)^. These established eating behaviours remain relatively stable over time; therefore, it is crucial to ensure children develop healthy eating behaviours from the outset^(1)^. Healthy eating behaviours are defined in this paper based on an understanding of intuitive eating: specifically eating for the purpose of maintaining health and energy and performing daily functions, rather than for appearance or weight-based motives^(9)^.

Concerningly, the media that young people consume is saturated with the promotion of ‘diet culture’, which encourages the restriction of food, idealisation of thinness and weight loss and obsession over appearance and weight where the ‘thin ideal’ is portrayed as the key indicator of an individual’s health, beauty and self-worth^(2,8)^. The highly profitable diet industry (worth over $66 billion) has a strong influence over social media content on Instagram, directly and surreptitiously encouraging the diet culture and appearance-focused messages through influencers and marketing^(5,10)^. Research has found that people who go on a diet with appearance-focused motives are more likely to engage in disordered eating behaviours^(11)^. This is concerning as childhood and adolescence are critical periods of growth that require a certain amount of nutrition for optimal development, which dieting and disordered eating behaviours inhibit^(8)^.

## TikTok and dieting

TikTok has become one of the most popular social media platforms in the world, with over 1·6 billion users^(12)^. Approximately 14 % of these users are under 18 years old^(12)^. TikTok’s popular trends involving music and comedy have a powerful influence over young people and encourages mimetic behaviours, thereby warranting investigation of the potential behavioural influence it has on young people’s eating behaviours^(13,14)^. A recent study highlighted how the eating behaviours portrayed on TikTok are focused on weight loss, with over 44 % of the videos analysed promoting weight loss and diet culture^(2)^. Furthermore, the role of dieting in achieving an idealised perception of health was overemphasised, and the video sample lacked nutrition education from experts^(2)^. Content that claimed to be ‘healthy eating’ contained inherent guilt-inducing messages through the moralisation of food and stigmatisation of certain body shapes and sizes, which may serve to normalise disordered eating behaviours^(15)^. Additionally, disordered eating behaviours may in fact be promoted through TikTok’s algorithm, which tailors content based on what a user has shown interest in, thus potentially distorting reality and exacerbating existing predispositions for body image issues^(2)^. Adolescents who are constantly exposed to health and beauty ideals through the media report feeling pressured to change their bodies to reflect these ideals, which may include poor and potentially harmful dietary choices and eating behaviours^(8,16)^.

Whilst research has highlighted the negative influence that social media can have on eating behaviours and health^(17,18)^, it has also proven to be a powerful and engaging platform that has the potential to spread positive and evidence-based information to its users^(19–23)^. From a public health perspective, TikTok presents a novel, cost-effective opportunity to widely distribute accurate and credible health information. In fact, adolescents are more likely to access and engage with health information on social media platforms than they are with traditional sources^(24)^. Considering TikTok’s surge in popularity, young user base and influence on users, research exploring the type of content relating to health behaviours is warranted. One such study explored what nutrition-related content people wanted to see on TikTok and why, resulting in a list of recommendations for dietitians to use for health promotion via social media^(23)^. However, there is limited research that focuses on the influence that dieting videos on TikTok may have on disordered eating amongst young people, including the risks and opportunities from a public health perspective. Intuitive eating principles and the critical appraisal of the thin ideal are crucial strategies for promoting positive body image and healthy eating behaviours in order to prevent eating disorders, thereby should be incorporated into nutrition health promotion^(25,26)^.

The present study aims to expand upon this area of TikTok research by investigating the eating behaviours portrayed on TikTok, with a particular focus on how this may affect children and adolescents who are a particularly vulnerable group at risk of eating disorders. These insights may inform the public health field about the potential strengths and harms associated with nutrition and dieting messages on social media.

## Methods

A descriptive content analysis of videos on TikTok relating to dieting and eating behaviours was conducted following the protocol developed by Mandzufas *et al.* ^(27)^. The most popular relevant hashtags were identified for analysis at the time of data collection (September 2022) by typing in the word ‘diet’ and the trending phrase ‘what I eat in a day’ in the TikTok mobile app hashtag search and then selecting those with the highest number of views which contained a majority of English language videos. The top hashtags were #diet (21·5 billion views), #whatieatinaday (13·4 billion views), #wieiad (2·0 billion views), #dietitian (1·6 billion views) and #diettips (1·2 billion views). The videos in the selected hashtags had a combined total of 39·7 billion views, proving their importance for investigation. The sample and time-sensitive metadata (such as likes, comments and shares) were collected on one day by screenshotting the video on the TikTok desktop web page and saving the screenshot and URL in a password-protected file to analyse at a later date. Duplicates were skipped, and the next video in order was collected instead. The sample had limited influence from the TikTok algorithm because an incognito window was used to prevent previous search history from interfering with results, the research was conducted on a ‘guest’ profile without entering any user details and the video search results on the hashtag page remained the same across multiple searches as they were ordered by most likes and views. The top fifty videos analysis (Table 1). This sample size was selected based on prior studies^(28)^ and after the piloting phase of the protocol^(27)^, in which the sample size was considered sufficiently large so that content patterns are evident. The codebook for this study was based on Mandzufas *et al.*’s protocol for analysing TikTok videos^(27)^. However, topic-specific variables, such as types of eating behaviours displayed, were inductively developed by and piloted by researchers (including the primary and secondary coder) coding ten randomly selected videos (*n* 2 from each hashtag). Further refinements of the codebook were made to reduce variability in coding, and another ten videos were coded by the researchers using the new codebook. After a few more minor refinements, researchers agreed that the codebook was sufficient for the primary and secondary coders to officially start coding the sample. The final topic-specific variables are defined in Table 2, expanding on the generic variables defined in the protocol by Mandzufas *et al.* such as age, gender, expertise and instructional, as outlined in Table 3. In addition, humour was defined as when the video was deemed to have humourous intent, that is, to provoke laughter and provide amusement.

**Table 1:** Number of videos referencing body-related variables by hashtag

Codes	Total across all videos (*n* 250)		#Diet (*n* 50)		#Dietitian (*n* 50)		#DietTips (*n* 50)		#WhatIEatInADay (*n* 50)		#WIEIAD (*n* 50)		Examples
	*n*	%	*n*	%	*n*	%	*n*	%	*n*	%	*n*	%	
Weight measurement	22	8·8 %	5	10·0 %	6	12·0 %	8	16·0 %	2	4·0 %	1	2·0 %	‘What I eat to lose 16 kg’.
Mention of calories	22	8·8 %	7	14·0 %	2	4·0 %	9	18·0 %	0	0 %	4	8·0 %	‘This is one pint of ice cream, 1000 calories. And this is 8 cups of protein ice cream, 385 calories’.
Referencing body image	83	33·2 %	16	32·0 %	15	30·0 %	20	40·0 %	9	18·0 %	23	46·0 %	Man filming his shirtless body in the mirror at different timepoints. ‘This is my body one day of dieting. One week of dieting. Two weeks of dieting’.
Negative portrayal of body image	40	16·0 %	10	20·0 %	8	16·0 %	8	16·0 %	6	12·0 %	8	16·0 %	A man starts working out to look more like the muscular men on TV his partner was admiring.
Positive	42	16·8 %	6	12·0 %	7	14·0 %	11	22·0 %	3	6·0 %	15	30·0 %	After a full day of eating, woman is looking at her outfit using the phone as a mirror. ‘This outfit looks so good on me, I don’t care what you say, this dress looks so good on me’.
Included body checking	47	18·8 %	8	16·0 %	8	16·0 %	10	20·0 %	4	8·0 %	17	34·0 %	Girl posing in front of mirror with stomach exposed and filming herself.
Comparison of body between timepoints	16	6·4 %	6	12·0 %	0	0 %	4	8·0 %	1	2·0 %	5	10·0 %	‘Diet journey. 83 kg–59·6 kg’. Photos of a woman at different timepoints, with the weight at the time in text.

**Table 2:** Topic-specific codebook with examples from the sample

Code	Definition	Example
Diet	Any content explicitly indicating that the creator participates in specific eating patterns with a particular goal behind it, such as losing weight or gaining muscle – not just everyday eating behaviours.	A compilation of clips about the creator’s weight loss during his diet. The creator says whilst filming his body in the mirror: ‘This is my body- one day of dieting. One week of dieting. Two weeks of dieting. Three weeks of dieting. One month of dieting’.
Type of diet	Any content that references or portrays a specific diet, such as vegan, low calorie, fasting, keto, etc.	‘What I ate today in a calorie deficit’.
Goal of diet	Creator explicitly indicates the goal of their diet: Weight loss Weight gain Muscle gain/performance enhancement for sport/fitness	Example of weight loss: ‘Healthy snack ideas from someone who lost 115 lbs in 10 months’. Example of weight gain and muscle gain: Creator explains how to make a protein smoothie. ‘Drink this every day to gain weight and muscle’.
Culinary instruction	Video teaches viewers how to make a recipe. Ingredients don’t necessarily need to be stated, but the video demonstrates how to cook something.	Video with voiceover instruction and visual demonstration of how to make breakfast burritos.
Type of eating behaviour	Any content that portrays or references: Disordered eating – includes a range of severe irregular eating behaviours (including restrictive and excessive) that indicate a diagnosis of a specific eating disorder. The eating behaviours must be clearly affecting the creator’s health or daily functioning, or the creator is modelling strategies or motivations for initiating disordered eating behaviours. Restricted and excessive eating – irregular eating behaviours but the effect on their health and daily functioning cannot be assumed, therefore not classed as disordered eating. Healthy eating behaviours – specifically eating for the purpose of maintaining health and energy and performing daily functions, rather than for appearance or weight-based motives.	Disordered eating: Video skit ‘if disordered eating had a voice’ where man tells himself to drink soft drinks when he’s hungry to avoid eating food. ‘Hey, I’m hungry’. ‘Just have a diet Coke, you’ll be fine’. Excessive eating: To add 34 lbs of pure muscle as Conor McGregor (UFC fighter) prepared for an upcoming fight, ‘he ate 6 meals a day’. Restricted eating: Woman telling viewers to only eat oats with water for breakfast for the next 5 d. ‘No milk, no cream, no sugar, no soda, no juices. Just put water!’ Healthy eating behaviours: ‘What I eat in a day as a fat girl who’s happy being her size, I guess. I eat when I’m hungry and that’s the most important thing’.
Weight measurement	Any content that refers to or portrays weight measurement.	‘Healthy snack ideas from someone who lost 115 lbs in 10 months’.
Calorie counting	Explicitly states a certain number of calories	Man explains what fast food he eats when on a diet with limited time and how many calories the food contains. ‘McDonalds McChicken, no mayo, 300 calories’.
Comparison of body across at least two timepoints	Common technique often involving body checking to compare the weight or appearance across at least two timepoints, for example, Day 1 of diet plan *v*. Day 20 of diet plan.	Text on screen that reads: ‘Me telling people it’s actually really simple to lose weight. For example’. Insert of before and after picture of someone’s weight loss.
Body image portrayed	Any content that refers to or portrays body image. Body image is how we think and feel about ourselves physically (including our perceived sexual attractiveness) and how we believe others see us. If body image is referenced or portrayed, is it in a positive or negative light?	Creator shares what she eats in a day after a binge with an eating disorder. She says ‘my body image is pretty bad so I’m gonna have a hard time eating this’. Text appears on the screen: ‘My binge was just sort of setting in at this point and I felt really badly about my body, but we pushed through’. Positive body image Creator shows her flat stomach with text saying ‘a body that looks like this’, then cuts to clip of her with a more rounded stomach and text ‘can also look like this’, suggesting realistic bodies are not always the ideal versions you see online and body shape can fluctuate. Negative body image Creator shows how a fitness model puts inserts in her clothing to make her body’s curves emphasised, creator insinuates these unrealistic beauty ideals are affecting her negatively.
Body checking	Body checking is the repeated checking of one’s shape or weight in the mirror or using the phone	Creator looking at her body in the mirror and hyper-analysing it.

**Table 3:** Generic codes obtained from the protocol by Mandzufas *et al.*

Code	Definition
Age	‘Indistinguishable’ if the face is not pictured or clear and cannot be determined otherwise. A person will be characterised as a ‘child’ only if they are clearly a child less than 18 years. If there is some ambiguity, they will be characterised as ‘adult’.
Gender	If the gender is stated (text or audio), characterise that person as to that gender. If not stated, choose the perception of gender presentation based on physical characteristics, dress and other features.
Instructional	The video demonstrates and/or explicitly states how to do something or informs viewers about any subject either verbally, textually or visually.
Expertise	‘Yes’: the person leading the instruction or education makes a statement in text or audio that they have a qualification or professional standing that would lead the viewer to believe they are an authority on the subject. Reputational claim examples: health coach, psychologist, researcher.

EM (primary coder) and GW (secondary coder) first conducted inter-rater reliability testing on the codebook to quantify the consistency of coding between the two coders, resulting in 84·7 % agreement across a random sample of five videos per hashtag (*n* 25), after which the codebook was then further clarified. EM then coded all 250 videos using Research Electronic Data Capture (REDCap)^(29,30)^, a secure web-based software platform hosted at the The Kids Research Institute Australia. The second round of inter-rater reliability testing between EM and GW was conducted on a different random sample of twenty-five videos to the first round, which produced a score of 91·3 % agreement, indicating that the video sample was coded consistently. The total number of videos coded by GW (secondary coder) for the purpose of consistency checking from both rounds was *n* 50. As stated in the protocol^(27)^, with the entire sample coded by EM (primary coder), and both rounds of inter-rater reliability testing scoring at least 80 % agreement, the consistency of coding was considered acceptable to perform data analysis.

R statistical software was used to perform descriptive data summaries of the variables coded. As engagement variables did not satisfy normality tests, a two-sample Mann–Whitney *U* test was used to compare the level of engagement videos received depending on the presence of certain variables. This test was performed at the significance value of *P* < 0·05. The following variables were investigated for interaction and significance based on existing literature on engaging audiences in social media and the aims of this study: video length, the inclusion of disordered eating, credibility and titles of the poster and the use of humour in the video. For techniques found to be significant, effect sizes were determined using Cliff’s delta. In addition, gender differences between types of diet were tested using a *χ* ^2^ test of independence at the significance value of <0·05.

## Results

Overall, the 250 videos contributed to a total of 197·2 million likes (*M* = 789 051·0, s d = 923 335·0), 1 267 251 comments (*M* = 5069·0, s d = 9315·3) and 3·8 million shares (*M* = 15 423·2, s d = 41 271·3). Most videos featured adults (*n* 205, 82 %), and only a small number of videos featured identifiable children or adolescents (*n* 16, 6·4 %). The information and advice regarding diets and eating behaviours came from two types of sources: ‘experts’ and regular creators sharing their personal experiences. The results showed that 16 % (*n* 40) of the videos had a blue tick verification, which indicates the creator’s social influencer status. On the contrary, users who mentioned their personal experience partaking in a diet made up more than half of the videos (*n* 154, 61·6 %). Furthermore, health conditions were mentioned in some videos (*n* 37, 14·8 %), of which 17 (45·9 %) related to eating disorders. Table 4 outlines the number of videos showcasing eating behaviours, including disordered eating, restricted eating, excessive eating and healthy eating behaviours. Other conditions mentioned were not relevant to the research question but encompass chronic conditions such as coeliac disease, irritable bowel syndrome, diabetes and several forms of cancer.

**Table 4:** Number of videos portraying eating behaviour categories by hashtag

Codes	Total across all videos (*n* 250)		#Diet (*n* 50)		#Dietitian (*n* 50)		#DietTips (*n* 50)		#WhatIEatInADay (*n* 50)		#WIEIAD (*n* 50)		Examples
	*n*	%	*n*	%	*n*	%	*n*	%	*n*	%	*n*	%	
Disordered eating	16	6·4 %	2	4·0 %	4	8·0 %	3	6·0 %	4	8·0 %	3	6·0 %	‘You can’t keep crunchy, salty carb snacks in the house without finishing the whole bag and feeling guilty’.
Excessive eating	9	3·6 %	3	6·0 %	0	0 %	2	4·0 %	1	2·0 %	3	6·0 %	‘Get that Caniac combo, 2200 calories. I’m on a bulk right now, gotta get those calories in’.
Healthy eating	136	54·4 %	16	32·0 %	32	64·0 %	15	30·0 %	40	80·0 %	33	66·0 %	‘Office workers eating more than 1200 calories because they feel more energised. People following a BALANCED DIET instead of restricted diets for long-term results’.
Restricted eating	26	10·4 %	5	10·0 %	6	12·0 %	7	14·0 %	2	4·0 %	6	12·0 %	‘How does Jennifer Lopez look this good at 51? Well according to her trainer she’s only allowed 1400 calories per day. For breakfast she has a smoothie, for lunch she has a salad, and for dinner grilled chicken’.
Other	74	29·6 %	26	52·0 %	13	26·0 %	24	48·0 %	4	8·0 %	7	14·0 %	Video did not specifically discuss or portray eating behaviours, such as videos of people at the gym or showing a food recipe.

Almost half of the videos were educational or instructional (*n* 116, 46·4 %), of which seventy-four (63·8 %) were posted by users who claimed to be experts in relation to the diet they were advising on. Furthermore, ‘dietitian’ was the most common title used amongst ‘experts’ (*n* 43, 58·1 %), followed by ‘health and wellness coach’ (*n* 7, 9·5 %), ‘doctor’ (MD; *n* 6, 8·1 %), ‘fitness trainer’ (*n* 6, 8·1 %) and ‘nutritionist’ (*n* 3, 4·1 %). Nutrition advice provided in the videos included recipe ideas (e.g. how to build a balanced meal with enough protein, healthy fats and fibre) or videos of what the creator ate to achieve a certain physique (e.g. for weight loss). Of the videos that specifically mentioned a certain diet, the most common diets were low calorie (*n* 32, 12·8 %), vegan (*n* 14, 5·6 %), self-reported healthy eating (*n* 12, 4·8 %) and fasting (*n* 10, 4·0 %). Other diets specifically mentioned were detox diets (such as a juice cleanse) (*n* 5, 2 %), diets for medical reasons (such as allergies or coeliac) (*n* 4, 1·6 %), keto (*n* 5, 2 %), low carbohydrate diets (*n* 8, 3·2 %), low-fat diets (*n* 7, 2·8 %), vegetarian diets (*n* 2, 0·8 %), non-specific diets (such as stating ‘I’m on a diet’) (*n* 28, 11·2 %), fad diets or food-specific diets (such as meat only diets) (*n* 13, 5·2 %) and ‘other’ diets (including increased protein, caloric surplus, whole30, paleo, weight watchers) (*n* 23, 9·2 %). References to body image in relation to the diets portrayed are presented in Table 1.

Humour was the only variable found to significantly impact engagement. The median (and IQR) of likes for videos with humour (*n* 64) *v*. not (*n* 186) are 764 050 (7 500 000) and 482 500 (515 000), respectively. The Mann–Whitney *U* test showed a significant difference between these two medians (*z* = 2·54, *P* = 0·011), and the subsequent cliff’s *d* showed a small effect size (*d* = 021; 95 % CI: 0·035, 0·38). Similarly, there was a significant difference between the medians of comments for videos with and without humour, 3252 (7074) and 1694 (4205), respectively (*z* = 2·06, *P* = 0·039). The same was found for the number of shares 6685 (26 816) and 1982 (7435), respectively (*z* = 2·66, *P* = 0·0076). Disordered eating, provision of advice and the expert status of the creator providing the advice did not show a significant difference between the medians of engagement. Video length was also not shown to be correlated with engagement.

Male user-generated content was mostly used in reference to enhance gym performance. A *χ* ^2^ test of independence to compare the presence of diets for enhanced performance in the gym amongst female and male users showed that a significant interaction was found (*X* ^2^ = 5·78; *P* = 0·016; 95 % CI: –0·45, –0·035), where videos by female users are less likely to contain this diet (10·3 %) than male users (34·6 %). Surprisingly, a significant interaction was found (*X* ^2^ = 7·23; *P* = 0·007; 95 % CI: –0·31, –0·038) where videos by female users were less likely to specify their diet was motivated by weight loss (19·2 %) than male users (36·7 %).

## Discussion

This study examined how dieting and eating behaviours are portrayed on TikTok and the potential implications of these results for children and adolescents. Overall, more than half of the diet-related videos (57 %) portrayed body checking, which is the repeated checking and hyper-fixation of one’s body in the mirror or by using the phone camera. This type of content can be problematic, as it reinforces the notion that food should be used primarily for the manipulation of body weight and appearance. Potentially harmful social comparisons are often made by females viewing ‘fitspiration’ content that often includes body checking and modelling the thin ideal, thereby negatively affecting their body image and self-esteem^(25)^. Promotion of the ‘thin ideal’ was a prevalent theme in this sample of videos, which can be especially harmful to female adolescents who are more vulnerable to the underlying societal pressures for appearance-focused diets^(11,31)^. Examples of the thin ideal being promoted within the sample were noted within videos that portrayed body checking, such as videos with text encouraging viewers to ‘become this girl with me’ over pictures of young women in gym clothes or everyday wear posing in front of mirrors and exposing their flat stomachs. On the contrary, themes of body positivity and body diversity were also prevalent. For example, some creators made statements such as ‘what I eat in a day as a fat person that is not focused on weight loss’, and some videos specifically called out the thin ideal and harmful comparisons online by stating ‘comparing yourself to another person’s diet is not helpful. Even if we all ate the same exact way every day, we would all look different. We all have our different needs and different preferences and that is totally okay.’

Over half (54·4 %) of the videos did exhibit a certain degree of healthy eating behaviours, and only 6·4 % represented disordered eating behaviours. However, it is important to note that although disordered eating behaviours were only present in a relatively small sample of videos as categorised by hashtags, when a user engages with these videos in their ‘For You page’, the algorithm will likely continue exposing them to similar content. The videos coded as restricted eating (10·4 %) signify videos actively promoting weight loss and diet culture. Furthermore, the high prevalence of body checking, alongside the portrayal of body image, highlights the tendency for social comparisons and the perceived link between food and self-worth on TikTok. An overabundance of ‘healthy’ eating messaging can lead to a culture of idealistic ways of eating and expose viewers to inherent guilt-inducing messages about food consumption^(15)^. Furthermore, this could contribute to increasing unrealistic social pressures around constantly being ‘healthy’ amongst young people and might prompt a hyper-fixation on food and nutrition^(9,15)^.

The combined engagement across all five hashtags shows that billions of viewers are interested in dieting and are potentially using TikTok as a source of information and advice about dieting. Nearly half (46·0 %) of the videos in this study provided some sort of nutrition advice, which is in contrast to a study on Instagram which found limited nutrition or exercise information within images under the hashtag #weightloss, with most focused on body appearance or images of food^(32)^. This reflects a possible difference between the two platforms, with higher amounts of users engaging with more information-based content on TikTok. Of the videos that provided nutrition advice, over half of the creators (64·0 %) claimed to have an expert qualification or status. More than half of the creators that claimed to be an expert, claimed to be a dietitian (58·1 %) and primarily gave instructions on what to cook and consume for weight loss or maintaining nutritious, yet low-calorie, diets. Those giving advice who did not specify a qualification do not necessarily provide lower quality advice; however, a study on Twitter found that non-professionals have been found to show false or misleading outcomes of diets which may contribute to misinformation^(33)^. Anyone can claim to be an expert on social media, and these claims may be used to boost one’s credibility and engagement. However, in this study, there was no significant association between claiming expertise and engagement. This study found that humour is more important in boosting a video’s engagement, with a significant association across the number of likes, comments and shares. This is consistent with other research investigating user’s perspectives of important video features, highlighting the importance of the creator’s persona including authenticity, relatability and humour^(13,23,34)^. For example, a recent study found that when videos used humour to communicate information and promote health advice, it enhanced the creator’s likeability and helped to engage a larger audience when spreading health information^(13)^. This provides a potential avenue whereby health professionals could reach a greater audience with evidence-based nutrition information in a way that users are receptive to. However, social media users should also be made aware that the primary purpose of social media such as TikTok is to entertain; therefore, educational videos may have limitations in communicating all the necessary information to the desired audience. Health information could accompany a disclaimer that individuals should seek personal health advice from their doctor before making any health decisions based on online content.

This study had a relatively small number of blue tick verified users, which suggests that social media influencers have less content domination in this topic than they do on other platforms, such as Instagram. This finding represents how the actual content is more important on TikTok than an individual’s number of followers, thus reducing barriers for health professionals in obtaining high engagement. However, a study on diet discourse on Twitter found that whilst the conversation was dominated by non-health professionals, there was also a large proportion of commercial interests and misinformation diluting the discourse, possibly as a way to market products and undermine public health action^(33)^. This highlights the need for strong personas on TikTok to disseminate evidence-based and body-positive information to counteract the negative effects that opposing forces may profit from. Additionally, media literacy education can be effective in building skills and confidence in adolescents to critically analyse media content and therefore resist social comparisons and the internalisation of the thin ideal^(35)^.

The demographics of the content creators revealed that the majority of videos featured adults. Children and adolescents who see these creators might be more inclined to trust adults giving them nutrition advice since many creators are presented as authority figures or likeable role models. The gender presentation of the content creators was also noted as many studies have shown how gender influences dieting behaviours, specifically how females are more likely to participate in dieting for weight loss than males^(26,36)^. However, this study revealed that the male creators were more likely to identify weight loss as the goal of their diet than females were (36·7 % and 19·2 %, respectively). Additionally, males were more likely (34·6 %) to disclose online that their diets were motivated by their fitness and/or for muscle gain purposes compared with females (10·3 %). This is a phenomenon that requires further investigation; however, it could be inferred that females are less willing to disclose their dieting motivations on such a public forum due to weight, which females are especially susceptible to^(11,31)^. Evidently, these findings highlight the need for evidence-based, positive eating behaviour content and education that aims to shift the focus from eating for your appearance to eating for nutrition, energy and enjoyment. Nutrition health promoters could utilise this evidence in conjunction with the recommendations provided by Shine *et al.* ^(13)^ to enhance their positive impact on young people.

### Limitations

This cross-sectional sample of videos represents the content at one point in time; however, the virality and rapidly changing nature of TikTok means that these findings may not apply to a later timepoint. Additionally, the accuracy of the nutrition information provided in the videos was not assessed. However, our findings are consistent with previous research and provide a valuable analysis of the dieting discourse on TikTok. Aspects of the analysis of this study are limited by the inability to accurately establish the age and gender of the individuals in each video based solely off physical attributes. Future research should include contacting the content creators of each analysed video to gather data on their age and the gender they identify as. As the eligibility criteria excluded non-English videos for analysis, the findings may not apply to non-Western cultures where the eating and dieting context can differ significantly. The design of this study relied on creating a consistent codebook to categorise the information, although the content is subjective and has the potential for observer bias. This was accounted for by conducting several rounds of inter-rater reliability testing and further refinement, but there is still the chance of bias due to the nature of the topic. The codebook specified definitions for identifying the variables; however, variables such as humour are inherently subjective. The research topic was categorised by hashtags for the purposes of the research design but does not represent the standard user experience when scrolling through the For You page. Nevertheless, information searching by hashtag on social media is not uncommon, especially for people with particular interests or belonging to subgroups such as those with eating disorders.

Another consideration for this study is that high video engagement (likes, comments and shares) does not necessarily mean the video is perceived as effective or likeable by the target audience. The comment section could contain negative comments or disagreement, which was not in the scope of analysis for this study. Future research should consider conducting a sentiment analysis on the comments of all videos sampled, as comments can provide critical information to inform content analyses of social media content. Similarly, we cannot assume the effect of these videos on viewers despite prior research indicating there is a link between watching behaviours and replicating those behaviours. As a novel platform, future research should track viewer’s behaviours after watching TikTok videos and analyse the types of techniques and messaging that are highly associated with behaviour change to inform public health action.

### Conclusion

This study aimed to investigate how dieting is portrayed on TikTok and the potential implications for public health considering the effect of diet culture on eating disorders amongst young people. The study provides a comprehensive snapshot of the representation and advice on dieting being widely disseminated on TikTok. The study found that creators employ a variety of techniques across their videos to share messages around diet and dieting. This brings a growing concern about the potentially harmful messaging from the persistent presence of diet culture on TikTok, mainly due to the large audience of vulnerable young viewers on the platform. However, positive messages were also prevalent within the sample, with examples of body positivity and healthy eating behaviours. Further research is required to investigate actual changes in viewer’s behaviours when exposed to both negative and positive health messaging on TikTok. However, these findings provide a valuable foundation and highlight a unique opportunity for health professionals to *positively* influence the public’s health behaviours by utilising the popularity of TikTok amongst young people to disseminate evidence-based information and promote healthy eating behaviours. Lastly, this study also highlights the variety of content available on social media and the need for media literacy education so viewers can critically analyse health information they are exposed to online.
